# {[(*N*-Butyl-*N*-methylcarbamothioyl)sulfanyl]acetato-κ*O*}tris(2-chloro­benz­yl)tin(IV)

**DOI:** 10.1107/S1600536810005830

**Published:** 2010-02-17

**Authors:** Thy Chun Keng, Kong Mun Lo, Seik Weng Ng

**Affiliations:** a Department of Chemistry, University of Malaya, 50603 Kuala Lumpur, Malaysia

## Abstract

The Sn atom in the title compound, [Sn(C_7_H_6_Cl)_3_(C_8_H_14_NO_2_S_2_)], is coordinated by three chlorobenzyl ligands and one carboxylate O atom of the substituted acetate ligand in a distorted tetra­hedral environment. Three of the C atoms of the *n*-butyl group are disordered over two sites with equal occupancies.

## Related literature

Trialkyl­tin carboxyl­ates are generally carboxyl­ate-bridged polymers; see: Ng *et al.* (1988 ▶). For the direct synthesis of substituted tribenzyl­tin chlorides, see: Sisido *et al.* (1961 ▶). For the synthesis of dithio­carbamoylacetic acids, see: Nachmias (1952 ▶). For background to the triorganotin derivatives of dithio­carbamylacetic acids, see: Ng & Kumar Das (1991 ▶).
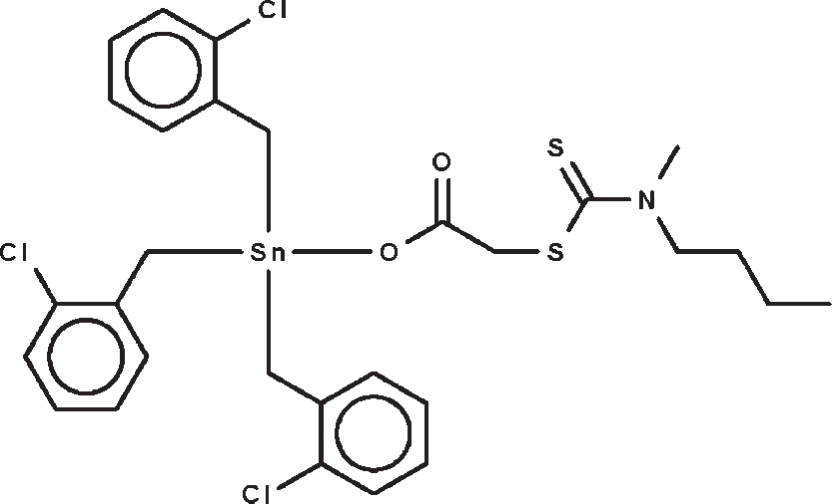


## Experimental

### 

#### Crystal data

[Sn(C_7_H_6_Cl)_3_(C_8_H_14_NO_2_S_2_)]
*M* *_r_* = 715.72
Triclinic, 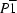

*a* = 10.2485 (2) Å
*b* = 11.7943 (2) Å
*c* = 13.1704 (3) Å
α = 84.417 (1)°
β = 84.113 (1)°
γ = 86.387 (1)°
*V* = 1573.79 (5) Å^3^
*Z* = 2
Mo *K*α radiation
μ = 1.23 mm^−1^
*T* = 293 K
0.30 × 0.20 × 0.10 mm

#### Data collection

Bruker SMART APEX diffractometer
Absorption correction: multi-scan (*SADABS*; Sheldrick, 1996 ▶) *T*_min_ = 0.710, *T*_max_ = 0.887
11006 measured reflections
6949 independent reflections
5708 reflections with *I* > 2σ(*I*)
*R*_int_ = 0.023

#### Refinement

*R*[*F* ^2^ > 2σ(*F* ^2^)] = 0.034
*wR*(*F* ^2^) = 0.078
*S* = 1.03
6949 reflections
353 parameters
10 restraints
H-atom parameters constrained
Δρ_max_ = 0.49 e Å^−3^
Δρ_min_ = −0.69 e Å^−3^

### 

Data collection: *APEX2* (Bruker, 2009 ▶); cell refinement: *SAINT* (Bruker, 2009 ▶); data reduction: *SAINT* program(s) used to solve structure: *SHELXS97* (Sheldrick, 2008 ▶); program(s) used to refine structure: *SHELXL97* (Sheldrick, 2008 ▶); molecular graphics: *X-SEED* (Barbour, 2001 ▶); software used to prepare material for publication: *publCIF* (Westrip, 2010 ▶).

## Supplementary Material

Crystal structure: contains datablocks global, I. DOI: 10.1107/S1600536810005830/lh2995sup1.cif

Structure factors: contains datablocks I. DOI: 10.1107/S1600536810005830/lh2995Isup2.hkl

Additional supplementary materials:  crystallographic information; 3D view; checkCIF report

## Figures and Tables

**Fig. 1.**
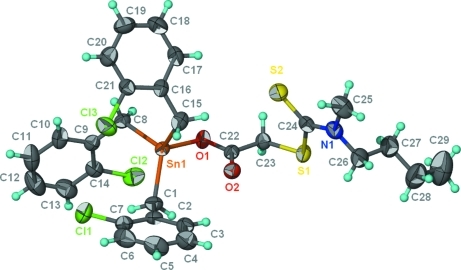
Thermal ellipsoid plot (Barbour, 2001) of Sn(C7H6Cl)3(C8H14N2O2S2) at the 50% probability level. Hydrogen atoms are drawn as spheres of arbitrary radius. The disorder in the butyl chain is not shown.

## supplementary crystallographic information

### Comment

The molecular structure of the title compound is shown in Fig. 1.

### Experimental

*n*-Butylmethyldithiocarbomylacetic acid was synthesized from *n*-butylmethylamine, carbon disulfide and chloroacetic acid (Nachmias, 1952). Tri(*o*-chlorobenzyl)tin chloride was prepared by direct synthesis from *o*-chlorobenzyl chloride and tin powder in a mixture of toluene and water (Sisido *et al.*, 1961). The triorganotin chloride was hydrolyzed with dilute sodium hydroxide solution to give tri(*o*-chlorobenzyl)tin hydroxide. The carboxylic acid (0.1 g, 0.45 mmol) and the organotin hydroxide (0.23 g, 0.45 mmol) were heated in ethanol (100 ml) for 1 hour. After filtering the mixture, colorless crystals were obtained upon slow evaporation of the filtrate.

### Refinement

Hydrogen atoms were placed at calculated positions (C–H 0.93–0.97 Å) and were treated as riding on their parent atoms, with *U*(H) set to 1.2–1.5 times *U*_eq_(C).

The butyl chain is disordered over two positions in the three end carbon atoms. The occupancy could not be refined, and was assumed to be 0.5:0.5.  The 1,2-related distances were restrained to 1.54±0.01 Å and the 1,3-related ones to 2.51±0.01 Å. The temperature factors of the primed atoms were restrained to those of the unprimed ones.

### Crystal data

**Table:**

[Sn(C_7_H_6_Cl)_3_(C_8_H_14_NO_2_S_2_)]	*Z* = 2
*M**_r_* = 715.72	*F*(000) = 724
Triclinic, *P*1	*D*_x_ = 1.510 Mg m^−^^3^
Hall symbol: -P 1	Mo *K*α radiation, λ = 0.71073 Å
*a* = 10.2485 (2) Å	Cell parameters from 3482 reflections
*b* = 11.7943 (2) Å	θ = 2.4–24.9°
*c* = 13.1704 (3) Å	µ = 1.23 mm^−^^1^
α = 84.417 (1)°	*T* = 293 K
β = 84.113 (1)°	Block, colorless
γ = 86.387 (1)°	0.30 × 0.20 × 0.10 mm
*V* = 1573.79 (5) Å^3^	

### Data collection

**Table:**

Bruker SMART APEX diffractometer	6949 independent reflections
Radiation source: fine-focus sealed tube	5708 reflections with *I* > 2σ(*I*)
graphite	*R*_int_ = 0.023
ω scans	θ_max_ = 27.5°, θ_min_ = 1.6°
Absorption correction: multi-scan (*SADABS*; Sheldrick, 1996)	*h* = −13→13
*T*_min_ = 0.710, *T*_max_ = 0.887	*k* = −15→15
11006 measured reflections	*l* = −15→17

### Refinement

**Table:**

Refinement on *F*^2^	Primary atom site location: structure-invariant direct methods
Least-squares matrix: full	Secondary atom site location: difference Fourier map
*R*[*F*^2^ > 2σ(*F*^2^)] = 0.034	Hydrogen site location: inferred from neighbouring sites
*wR*(*F*^2^) = 0.078	H-atom parameters constrained
*S* = 1.03	*w* = 1/[σ^2^(*F*_o_^2^) + (0.032*P*)^2^ + 0.1764*P*] where *P* = (*F*_o_^2^ + 2*F*_c_^2^)/3
6949 reflections	(Δ/σ)_max_ = 0.001
353 parameters	Δρ_max_ = 0.49 e Å^−^^3^
10 restraints	Δρ_min_ = −0.69 e Å^−^^3^

### Fractional atomic coordinates and isotropic or equivalent isotropic displacement parameters (Å^2^)

**Table:**

	*x*	*y*	*z*	*U*_iso_*/*U*_eq_	Occ. (<1)
Sn1	0.204624 (18)	0.556101 (16)	0.639187 (15)	0.03536 (7)	
Cl1	0.34853 (10)	0.32147 (9)	0.86752 (8)	0.0813 (3)	
Cl2	0.14093 (9)	0.31598 (8)	0.51718 (7)	0.0639 (2)	
Cl3	0.14793 (11)	0.58144 (9)	0.91177 (8)	0.0797 (3)	
S1	0.40006 (8)	0.83118 (6)	0.30222 (6)	0.0476 (2)	
S2	0.22340 (9)	0.95231 (8)	0.45925 (7)	0.0589 (2)	
O1	0.18941 (19)	0.61434 (17)	0.48768 (15)	0.0440 (5)	
O2	0.38538 (19)	0.67862 (17)	0.50007 (15)	0.0447 (5)	
N1	0.4466 (3)	1.0273 (2)	0.3635 (2)	0.0517 (7)	
C1	0.3727 (3)	0.4388 (3)	0.6482 (2)	0.0460 (7)	
H1A	0.3428	0.3630	0.6699	0.055*	
H1B	0.4190	0.4366	0.5803	0.055*	
C2	0.4663 (3)	0.4680 (2)	0.7200 (2)	0.0418 (7)	
C3	0.5614 (3)	0.5462 (3)	0.6885 (3)	0.0490 (8)	
H3	0.5636	0.5823	0.6224	0.059*	
C4	0.6529 (4)	0.5726 (3)	0.7516 (3)	0.0645 (10)	
H4	0.7152	0.6254	0.7279	0.077*	
C5	0.6513 (4)	0.5205 (4)	0.8489 (3)	0.0723 (11)	
H5	0.7129	0.5374	0.8916	0.087*	
C6	0.5586 (4)	0.4433 (3)	0.8837 (3)	0.0673 (11)	
H6	0.5577	0.4074	0.9498	0.081*	
C7	0.4664 (3)	0.4186 (3)	0.8204 (2)	0.0498 (8)	
C8	0.0185 (3)	0.4777 (2)	0.6709 (2)	0.0393 (6)	
H8A	−0.0351	0.5175	0.7228	0.047*	
H8B	−0.0266	0.4866	0.6092	0.047*	
C9	0.0306 (3)	0.3539 (2)	0.7071 (2)	0.0393 (6)	
C10	−0.0111 (3)	0.3140 (3)	0.8071 (2)	0.0543 (8)	
H10	−0.0457	0.3660	0.8528	0.065*	
C11	−0.0028 (4)	0.1998 (4)	0.8405 (3)	0.0763 (12)	
H11	−0.0320	0.1752	0.9078	0.092*	
C12	0.0493 (5)	0.1219 (3)	0.7732 (4)	0.0847 (14)	
H12	0.0544	0.0445	0.7954	0.102*	
C13	0.0931 (4)	0.1575 (3)	0.6746 (3)	0.0668 (10)	
H13	0.1287	0.1051	0.6295	0.080*	
C14	0.0838 (3)	0.2722 (3)	0.6428 (2)	0.0464 (7)	
C15	0.2045 (3)	0.7154 (3)	0.7065 (2)	0.0503 (8)	
H15A	0.2742	0.7113	0.7515	0.060*	
H15B	0.2226	0.7761	0.6526	0.060*	
C16	0.0771 (3)	0.7435 (2)	0.7657 (2)	0.0402 (7)	
C17	−0.0152 (3)	0.8235 (3)	0.7272 (3)	0.0549 (8)	
H17	0.0037	0.8617	0.6627	0.066*	
C18	−0.1340 (4)	0.8476 (3)	0.7821 (3)	0.0659 (10)	
H18	−0.1932	0.9021	0.7545	0.079*	
C19	−0.1655 (4)	0.7923 (3)	0.8764 (3)	0.0666 (10)	
H19	−0.2455	0.8094	0.9132	0.080*	
C20	−0.0780 (3)	0.7112 (3)	0.9169 (3)	0.0571 (9)	
H20	−0.0985	0.6728	0.9810	0.068*	
C21	0.0404 (3)	0.6876 (2)	0.8614 (2)	0.0431 (7)	
C22	0.2903 (3)	0.6740 (2)	0.4527 (2)	0.0369 (6)	
C23	0.2751 (3)	0.7353 (2)	0.3481 (2)	0.0416 (7)	
H23A	0.1908	0.7777	0.3505	0.050*	
H23B	0.2739	0.6789	0.2994	0.050*	
C24	0.3591 (3)	0.9463 (2)	0.3791 (2)	0.0427 (7)	
C25	0.4238 (4)	1.1315 (3)	0.4164 (3)	0.0721 (11)	
H25A	0.4044	1.1116	0.4886	0.108*	
H25B	0.3511	1.1764	0.3903	0.108*	
H25C	0.5011	1.1748	0.4051	0.108*	
C26	0.5641 (3)	1.0227 (3)	0.2910 (3)	0.0594 (9)	
H26A	0.5949	0.9436	0.2868	0.071*	0.50
H26B	0.6326	1.0619	0.3166	0.071*	0.50
H26C	0.5873	0.9438	0.2787	0.071*	0.50
H26D	0.6365	1.0517	0.3209	0.071*	0.50
C27	0.541 (3)	1.076 (3)	0.1840 (11)	0.070 (3)	0.50
H27A	0.5063	1.1541	0.1878	0.083*	0.50
H27B	0.4774	1.0340	0.1558	0.083*	0.50
C28	0.670 (3)	1.075 (3)	0.1132 (11)	0.097 (3)	0.50
H28A	0.7269	1.1307	0.1323	0.116*	0.50
H28B	0.7153	1.0004	0.1214	0.116*	0.50
C29	0.644 (5)	1.103 (3)	0.0016 (9)	0.140 (8)	0.50
H29A	0.7228	1.0885	−0.0420	0.210*	0.50
H29B	0.6150	1.1818	−0.0094	0.210*	0.50
H29C	0.5766	1.0560	−0.0141	0.210*	0.50
C27'	0.545 (3)	1.091 (3)	0.1895 (11)	0.070 (3)	0.50
H27C	0.5421	1.1721	0.1988	0.083*	0.50
H27D	0.4623	1.0743	0.1665	0.083*	0.50
C28'	0.658 (3)	1.064 (3)	0.1076 (11)	0.097 (3)	0.50
H28C	0.7411	1.0656	0.1358	0.116*	0.50
H28D	0.6498	0.9875	0.0880	0.116*	0.50
C29'	0.654 (5)	1.149 (3)	0.0135 (10)	0.140 (8)	0.50
H29D	0.7279	1.1336	−0.0348	0.210*	0.50
H29E	0.6571	1.2251	0.0335	0.210*	0.50
H29F	0.5741	1.1431	−0.0175	0.210*	0.50

### Atomic displacement parameters (Å^2^)

**Table:**

	*U*^11^	*U*^22^	*U*^33^	*U*^12^	*U*^13^	*U*^23^
Sn1	0.03237 (11)	0.03689 (11)	0.03697 (11)	−0.00267 (7)	−0.00229 (8)	−0.00472 (8)
Cl1	0.0721 (6)	0.0797 (7)	0.0813 (7)	−0.0031 (5)	0.0255 (5)	0.0124 (5)
Cl2	0.0628 (5)	0.0761 (6)	0.0531 (5)	0.0057 (4)	0.0016 (4)	−0.0211 (4)
Cl3	0.0783 (7)	0.0837 (7)	0.0698 (6)	0.0212 (6)	−0.0118 (5)	0.0175 (5)
S1	0.0548 (5)	0.0414 (4)	0.0446 (4)	−0.0085 (4)	0.0098 (4)	−0.0048 (3)
S2	0.0646 (6)	0.0545 (5)	0.0544 (5)	0.0056 (4)	0.0069 (4)	−0.0086 (4)
O1	0.0434 (11)	0.0509 (12)	0.0382 (11)	−0.0141 (10)	−0.0055 (9)	0.0025 (9)
O2	0.0377 (11)	0.0518 (13)	0.0451 (12)	−0.0035 (9)	−0.0086 (10)	−0.0018 (10)
N1	0.0565 (17)	0.0378 (14)	0.0614 (18)	−0.0043 (12)	−0.0096 (14)	−0.0032 (12)
C1	0.0373 (16)	0.0455 (17)	0.0560 (19)	0.0023 (13)	−0.0082 (14)	−0.0074 (14)
C2	0.0348 (15)	0.0441 (17)	0.0462 (18)	0.0068 (13)	−0.0048 (13)	−0.0078 (14)
C3	0.0468 (18)	0.0492 (18)	0.0508 (19)	−0.0010 (15)	−0.0080 (15)	−0.0016 (15)
C4	0.055 (2)	0.060 (2)	0.082 (3)	−0.0074 (17)	−0.022 (2)	−0.006 (2)
C5	0.073 (3)	0.078 (3)	0.074 (3)	0.004 (2)	−0.039 (2)	−0.015 (2)
C6	0.082 (3)	0.074 (3)	0.045 (2)	0.018 (2)	−0.016 (2)	−0.0052 (18)
C7	0.0459 (18)	0.053 (2)	0.0474 (19)	0.0085 (15)	0.0000 (15)	−0.0025 (15)
C8	0.0331 (14)	0.0414 (16)	0.0442 (17)	−0.0026 (12)	−0.0032 (13)	−0.0086 (13)
C9	0.0338 (15)	0.0433 (16)	0.0426 (17)	−0.0076 (12)	−0.0079 (13)	−0.0057 (13)
C10	0.059 (2)	0.061 (2)	0.0442 (18)	−0.0208 (17)	−0.0066 (16)	0.0004 (16)
C11	0.088 (3)	0.081 (3)	0.061 (2)	−0.037 (2)	−0.019 (2)	0.021 (2)
C12	0.101 (3)	0.048 (2)	0.109 (4)	−0.019 (2)	−0.039 (3)	0.012 (2)
C13	0.070 (2)	0.046 (2)	0.088 (3)	−0.0021 (18)	−0.023 (2)	−0.012 (2)
C14	0.0458 (17)	0.0434 (17)	0.0521 (19)	−0.0043 (14)	−0.0121 (15)	−0.0063 (14)
C15	0.0547 (19)	0.0476 (18)	0.0502 (19)	−0.0136 (15)	0.0046 (15)	−0.0148 (15)
C16	0.0487 (17)	0.0340 (15)	0.0398 (16)	−0.0109 (13)	−0.0014 (13)	−0.0098 (12)
C17	0.070 (2)	0.0461 (19)	0.0468 (19)	−0.0034 (17)	−0.0067 (17)	0.0046 (15)
C18	0.064 (2)	0.057 (2)	0.075 (3)	0.0117 (18)	−0.014 (2)	0.0041 (19)
C19	0.048 (2)	0.072 (3)	0.077 (3)	0.0065 (18)	0.0035 (19)	−0.012 (2)
C20	0.061 (2)	0.068 (2)	0.0411 (18)	−0.0057 (18)	0.0018 (16)	−0.0061 (16)
C21	0.0488 (18)	0.0421 (17)	0.0386 (16)	0.0002 (13)	−0.0068 (14)	−0.0038 (13)
C22	0.0368 (15)	0.0360 (15)	0.0382 (16)	−0.0021 (12)	−0.0009 (13)	−0.0075 (12)
C23	0.0445 (17)	0.0417 (16)	0.0391 (16)	−0.0054 (13)	−0.0062 (13)	−0.0020 (13)
C24	0.0523 (18)	0.0371 (16)	0.0387 (16)	0.0017 (13)	−0.0095 (14)	−0.0006 (13)
C25	0.095 (3)	0.044 (2)	0.083 (3)	−0.0074 (19)	−0.023 (2)	−0.0156 (19)
C26	0.049 (2)	0.053 (2)	0.076 (3)	−0.0141 (16)	−0.0088 (18)	0.0018 (18)
C27	0.064 (3)	0.069 (6)	0.074 (3)	−0.014 (3)	−0.007 (2)	0.007 (3)
C28	0.072 (6)	0.125 (7)	0.090 (4)	−0.025 (4)	−0.003 (4)	0.014 (3)
C29	0.153 (10)	0.17 (3)	0.094 (5)	−0.051 (17)	0.005 (7)	0.016 (8)
C27'	0.064 (3)	0.069 (6)	0.074 (3)	−0.014 (3)	−0.007 (2)	0.007 (3)
C28'	0.072 (6)	0.125 (7)	0.090 (4)	−0.025 (4)	−0.003 (4)	0.014 (3)
C29'	0.153 (10)	0.17 (3)	0.094 (5)	−0.051 (17)	0.005 (7)	0.016 (8)

### Geometric parameters (Å, °)

**Table:**

Sn1—O1	2.0643 (19)	C15—H15A	0.9700
Sn1—C1	2.146 (3)	C15—H15B	0.9700
Sn1—C15	2.154 (3)	C16—C21	1.391 (4)
Sn1—C8	2.157 (3)	C16—C17	1.392 (4)
Cl1—C7	1.743 (4)	C17—C18	1.379 (5)
Cl2—C14	1.738 (3)	C17—H17	0.9300
Cl3—C21	1.743 (3)	C18—C19	1.363 (5)
S1—C24	1.775 (3)	C18—H18	0.9300
S1—C23	1.782 (3)	C19—C20	1.376 (5)
S2—C24	1.659 (3)	C19—H19	0.9300
O1—C22	1.307 (3)	C20—C21	1.378 (4)
O2—C22	1.217 (3)	C20—H20	0.9300
N1—C24	1.339 (4)	C22—C23	1.510 (4)
N1—C26	1.460 (4)	C23—H23A	0.9700
N1—C25	1.466 (4)	C23—H23B	0.9700
C1—C2	1.491 (4)	C25—H25A	0.9600
C1—H1A	0.9700	C25—H25B	0.9600
C1—H1B	0.9700	C25—H25C	0.9600
C2—C3	1.389 (4)	C26—C27'	1.518 (8)
C2—C7	1.392 (4)	C26—C27	1.521 (8)
C3—C4	1.383 (4)	C26—H26A	0.9700
C3—H3	0.9300	C26—H26B	0.9700
C4—C5	1.365 (5)	C26—H26C	0.9700
C4—H4	0.9300	C26—H26D	0.9700
C5—C6	1.371 (5)	C27—C28	1.537 (9)
C5—H5	0.9300	C27—H27A	0.9700
C6—C7	1.384 (5)	C27—H27B	0.9700
C6—H6	0.9300	C28—C29	1.522 (10)
C8—C9	1.492 (4)	C28—H28A	0.9700
C8—H8A	0.9700	C28—H28B	0.9700
C8—H8B	0.9700	C29—H29A	0.9600
C9—C10	1.389 (4)	C29—H29B	0.9600
C9—C14	1.393 (4)	C29—H29C	0.9600
C10—C11	1.376 (5)	C27'—C28'	1.536 (9)
C10—H10	0.9300	C27'—H27C	0.9700
C11—C12	1.384 (6)	C27'—H27D	0.9700
C11—H11	0.9300	C28'—C29'	1.522 (10)
C12—C13	1.366 (6)	C28'—H28C	0.9700
C12—H12	0.9300	C28'—H28D	0.9700
C13—C14	1.377 (5)	C29'—H29D	0.9600
C13—H13	0.9300	C29'—H29E	0.9600
C15—C16	1.487 (4)	C29'—H29F	0.9600
			
O1—Sn1—C1	109.03 (10)	C19—C20—C21	119.3 (3)
O1—Sn1—C15	100.56 (11)	C19—C20—H20	120.3
C1—Sn1—C15	118.90 (12)	C21—C20—H20	120.3
O1—Sn1—C8	98.45 (10)	C20—C21—C16	122.8 (3)
C1—Sn1—C8	114.34 (11)	C20—C21—Cl3	118.8 (2)
C15—Sn1—C8	112.37 (11)	C16—C21—Cl3	118.3 (2)
C24—S1—C23	101.94 (14)	O2—C22—O1	123.0 (3)
C22—O1—Sn1	109.69 (17)	O2—C22—C23	124.4 (3)
C24—N1—C26	124.0 (3)	O1—C22—C23	112.6 (2)
C24—N1—C25	120.2 (3)	C22—C23—S1	114.9 (2)
C26—N1—C25	115.7 (3)	C22—C23—H23A	108.5
C2—C1—Sn1	113.91 (19)	S1—C23—H23A	108.5
C2—C1—H1A	108.8	C22—C23—H23B	108.5
Sn1—C1—H1A	108.8	S1—C23—H23B	108.5
C2—C1—H1B	108.8	H23A—C23—H23B	107.5
Sn1—C1—H1B	108.8	N1—C24—S2	124.3 (2)
H1A—C1—H1B	107.7	N1—C24—S1	113.1 (2)
C3—C2—C7	116.0 (3)	S2—C24—S1	122.57 (18)
C3—C2—C1	120.8 (3)	N1—C25—H25A	109.5
C7—C2—C1	123.2 (3)	N1—C25—H25B	109.5
C4—C3—C2	122.6 (3)	H25A—C25—H25B	109.5
C4—C3—H3	118.7	N1—C25—H25C	109.5
C2—C3—H3	118.7	H25A—C25—H25C	109.5
C5—C4—C3	119.6 (4)	H25B—C25—H25C	109.5
C5—C4—H4	120.2	N1—C26—C27'	112.5 (10)
C3—C4—H4	120.2	N1—C26—C27	113.3 (10)
C4—C5—C6	119.9 (4)	N1—C26—H26A	108.9
C4—C5—H5	120.0	C27'—C26—H26A	115.9
C6—C5—H5	120.0	C27—C26—H26A	108.9
C5—C6—C7	120.1 (4)	N1—C26—H26B	108.9
C5—C6—H6	120.0	C27'—C26—H26B	102.5
C7—C6—H6	120.0	C27—C26—H26B	108.9
C6—C7—C2	121.8 (3)	H26A—C26—H26B	107.7
C6—C7—Cl1	118.9 (3)	N1—C26—H26C	109.1
C2—C7—Cl1	119.2 (3)	C27'—C26—H26C	109.1
C9—C8—Sn1	113.64 (18)	C27—C26—H26C	101.9
C9—C8—H8A	108.8	N1—C26—H26D	109.1
Sn1—C8—H8A	108.8	C27'—C26—H26D	109.1
C9—C8—H8B	108.8	C27—C26—H26D	115.2
Sn1—C8—H8B	108.8	H26C—C26—H26D	107.8
H8A—C8—H8B	107.7	C26—C27—C28	110.7 (8)
C10—C9—C14	116.3 (3)	C26—C27—H27A	109.5
C10—C9—C8	121.5 (3)	C28—C27—H27A	109.5
C14—C9—C8	122.1 (3)	C26—C27—H27B	109.5
C11—C10—C9	121.9 (3)	C28—C27—H27B	109.5
C11—C10—H10	119.0	H27A—C27—H27B	108.1
C9—C10—H10	119.0	C29—C28—C27	110.9 (10)
C10—C11—C12	119.5 (4)	C29—C28—H28A	109.5
C10—C11—H11	120.3	C27—C28—H28A	109.5
C12—C11—H11	120.3	C29—C28—H28B	109.5
C13—C12—C11	120.6 (4)	C27—C28—H28B	109.5
C13—C12—H12	119.7	H28A—C28—H28B	108.1
C11—C12—H12	119.7	C28—C29—H29A	109.5
C12—C13—C14	118.9 (4)	C28—C29—H29B	109.5
C12—C13—H13	120.5	H29A—C29—H29B	109.5
C14—C13—H13	120.5	C28—C29—H29C	109.5
C13—C14—C9	122.8 (3)	H29A—C29—H29C	109.5
C13—C14—Cl2	118.3 (3)	H29B—C29—H29C	109.5
C9—C14—Cl2	119.0 (2)	C26—C27'—C28'	111.1 (8)
C16—C15—Sn1	112.23 (19)	C26—C27'—H27C	109.4
C16—C15—H15A	109.2	C28'—C27'—H27C	109.4
Sn1—C15—H15A	109.2	C26—C27'—H27D	109.4
C16—C15—H15B	109.2	C28'—C27'—H27D	109.4
Sn1—C15—H15B	109.2	H27C—C27'—H27D	108.0
H15A—C15—H15B	107.9	C29'—C28'—C27'	110.8 (9)
C21—C16—C17	115.8 (3)	C29'—C28'—H28C	109.5
C21—C16—C15	122.0 (3)	C27'—C28'—H28C	109.5
C17—C16—C15	122.2 (3)	C29'—C28'—H28D	109.5
C18—C17—C16	121.7 (3)	C27'—C28'—H28D	109.5
C18—C17—H17	119.1	H28C—C28'—H28D	108.1
C16—C17—H17	119.1	C28'—C29'—H29D	109.5
C19—C18—C17	120.7 (3)	C28'—C29'—H29E	109.5
C19—C18—H18	119.7	H29D—C29'—H29E	109.5
C17—C18—H18	119.7	C28'—C29'—H29F	109.5
C18—C19—C20	119.6 (3)	H29D—C29'—H29F	109.5
C18—C19—H19	120.2	H29E—C29'—H29F	109.5
C20—C19—H19	120.2		
			
C1—Sn1—O1—C22	−65.14 (19)	C1—Sn1—C15—C16	−136.0 (2)
C15—Sn1—O1—C22	60.61 (19)	C8—Sn1—C15—C16	1.4 (3)
C8—Sn1—O1—C22	175.38 (18)	Sn1—C15—C16—C21	74.5 (3)
O1—Sn1—C1—C2	126.2 (2)	Sn1—C15—C16—C17	−102.6 (3)
C15—Sn1—C1—C2	11.9 (3)	C21—C16—C17—C18	1.7 (5)
C8—Sn1—C1—C2	−124.7 (2)	C15—C16—C17—C18	179.0 (3)
Sn1—C1—C2—C3	−84.0 (3)	C16—C17—C18—C19	−0.7 (6)
Sn1—C1—C2—C7	97.2 (3)	C17—C18—C19—C20	−0.4 (6)
C7—C2—C3—C4	1.3 (4)	C18—C19—C20—C21	0.3 (6)
C1—C2—C3—C4	−177.6 (3)	C19—C20—C21—C16	0.9 (5)
C2—C3—C4—C5	0.0 (5)	C19—C20—C21—Cl3	−178.0 (3)
C3—C4—C5—C6	−0.5 (6)	C17—C16—C21—C20	−1.8 (4)
C4—C5—C6—C7	−0.4 (6)	C15—C16—C21—C20	−179.1 (3)
C5—C6—C7—C2	1.7 (5)	C17—C16—C21—Cl3	177.0 (2)
C5—C6—C7—Cl1	−179.1 (3)	C15—C16—C21—Cl3	−0.2 (4)
C3—C2—C7—C6	−2.1 (4)	Sn1—O1—C22—O2	9.4 (3)
C1—C2—C7—C6	176.8 (3)	Sn1—O1—C22—C23	−170.59 (17)
C3—C2—C7—Cl1	178.7 (2)	O2—C22—C23—S1	−7.0 (4)
C1—C2—C7—Cl1	−2.3 (4)	O1—C22—C23—S1	173.06 (19)
O1—Sn1—C8—C9	122.2 (2)	C24—S1—C23—C22	−73.4 (2)
C1—Sn1—C8—C9	6.8 (2)	C26—N1—C24—S2	−178.4 (2)
C15—Sn1—C8—C9	−132.6 (2)	C25—N1—C24—S2	−2.0 (4)
Sn1—C8—C9—C10	111.1 (3)	C26—N1—C24—S1	0.0 (4)
Sn1—C8—C9—C14	−69.0 (3)	C25—N1—C24—S1	176.5 (2)
C14—C9—C10—C11	−1.2 (5)	C23—S1—C24—N1	175.4 (2)
C8—C9—C10—C11	178.7 (3)	C23—S1—C24—S2	−6.2 (2)
C9—C10—C11—C12	0.4 (6)	C24—N1—C26—C27'	98.0 (16)
C10—C11—C12—C13	0.5 (6)	C25—N1—C26—C27'	−78.6 (16)
C11—C12—C13—C14	−0.5 (6)	C24—N1—C26—C27	89.5 (16)
C12—C13—C14—C9	−0.4 (5)	C25—N1—C26—C27	−87.1 (16)
C12—C13—C14—Cl2	179.7 (3)	N1—C26—C27—C28	176.6 (17)
C10—C9—C14—C13	1.2 (5)	C27'—C26—C27—C28	91 (14)
C8—C9—C14—C13	−178.6 (3)	C26—C27—C28—C29	167 (2)
C10—C9—C14—Cl2	−178.9 (2)	N1—C26—C27'—C28'	−167.3 (17)
C8—C9—C14—Cl2	1.3 (4)	C27—C26—C27'—C28'	−70 (13)
O1—Sn1—C15—C16	105.2 (2)	C26—C27'—C28'—C29'	−168 (2)
